# The rhizosphere Microbiome of *Malus sieversii* (Ldb.) Roem. in the geographic and environmental gradients of China's Xinjiang

**DOI:** 10.1186/s12866-023-02763-7

**Published:** 2023-01-21

**Authors:** Huiying Jiao, Liqiang Liu, Ruizhe Wang, Wei Qin, Bo Zhang

**Affiliations:** 1 Faculty of Horticulture, Xinjiang Agricultural University, Urumqi, 830052 China; 2 Faculty of Resources and Environment, Xinjiang Agricultural University, Urumqi, 830052 China

**Keywords:** Bacterial, Eukaryotic, Diversity, Community structure, Geographical distance, Mantel analysis

## Abstract

*Malus sieversii* (Ldb.) Roem. is the original species of modern cultivated apple and a key national essential conservation plant in China. In recent years, degradation and death of wild apple has been exacerbated by imbalances in the rhizosphere micro-ecosystems of wild apple forests due to soil nutrient loss, grazing, climate change and pest and disease outbreaks. However, the structure, diversity and response to environmental factors of wild apple rhizosphere microbial communities are so far unclear. In this study, the rhizosphere bacterial and eukaryotic communities of *M. sieversii* (Ldb.) Roem. in eight regions of the Yili River were analyzed using 16S/18S rDNA high-throughput sequencing technology. The results indicated that the bacterial operational taxonomic units (OTUs), Shannon index, and community composition were significantly lower in regions A, E, and F than in other regions. By contrast, the dominant eukaryotic communities in all regions were relatively similar in composition and differed less than the relative abundance of bacterial communities. Geographical and climatic distance were found to be key factors influencing the composition and diversity of wild apple rhizosphere microbial communities through mantel analysis. Moreover, these factors above were more correlated with bacterial diversity than with eukaryotes. This study identified the structure of wild apple rhizosphere microbial communities in Xinjiang and their interaction mechanisms under geographical and environmental gradients. It provides guidance for the sustainable management and ecological construction of wild apple forests in China.

## Introduction

*Malus sieversii* (Ldb.) Roem. was an important germplasm resource in China and is the main ancestral origin of cultivated apples [1, 2]. Belonging to Rosaceae (*Malus* Mill.), it is a Tertiary relict that has been included in the National Second-Class Protected Plant List and the China Plant Red Book [3]. This species was mainly distributed in the Tianshan Mountains across Central Asia. In China, it was found mostly in Xinyuan, Yining, Huocheng, and Gongliu counties in the Yili region of Xinjiang, as well as Emin and Tuoli counties in the Tacheng region [4]. The climatic environment of these regions is complex. Xinyuan, Gongliu, and Huocheng counties belonged to the trans-temperate continental and alpine climate. The Tacheng region, on the other hand, was in the mid-temperate arid and semiarid climate zones. In recent years, the pressure on the ecosystem was increasing due to soil nutrient loss, human grazing activities, climate change, and outbreaks of pest and disease [5, 6]. This led to the ecological fragility of the wild apple forestland, the loss of its original adaptive capacity, its large-scale degradation and even mortality.

Bacteria, archaea and eukaryotes in the rhizosphere microbial community were essential bioactive components of forest ecosystems [7]. It was found that maintaining the dynamic balance and diversity of the rhizosphere microbial community structure will helped to protect the co-ordination mechanisms of the whole ecosystem and buffered negative impacts [8]. Philippot et al. confirmed that the rate of the denitrification process decreases with the loss of soil microorganisms [9]. Many studies have shown that ecosystem processes are determined by the balance among species identity, community composition and species richness [10]. In addition, environmental factors that affect microbial community diversity and composition also affect ecosystem processes and functions [11]. Therefore, understanding the response patterns of wild apple rhizosphere microorganisms in different regions to complex environmental changes from the aspects of community composition, functional groups, and ecosystems is not only helpful for the protection of wild apple germplasm resources, but also can provide the theoretical basis for optimizing rhizosphere ecological environmental conditions of wild apple growth and decreasing the occurrence of soil-borne diseases.

There were many studies on the apple rhizosphere microbial community that mainly focus on biological control. Jiang et al. analyzed the rhizosphere microbial communities of apple trees around the Bohai Sea, and used high-throughput sequencing technology to explore the rhizosphere microbial communities of perennial and replanted apple trees in the Bohai Bay area, including bacteria and fungi [12]. The microbiota of the Lebanese wild apple (*Malus trilobata*) is a rich source of potential biocontrol agents for postharvest fungal pathogens in apples. Elie et al. explored the microbiota of wild apples (*M. trilobata*) as a potential source of two novel biocontrol agents for postharvest (*Botrytis cinerea* and *Penicillium expansum*) affecting commercial apples [13]. However, there are relatively fewer studies on the community structural analysis of rhizosphere bacteria and eukaryotes of wild apples in Xinjiang and the protection of germplasm resources.

In this study, 16S/18S rDNA analysis were conducted on the rhizosphere soil of wild apples in eight regions of Yili, Xinjiang, China. Further, the interaction of microbial community composition and diversity in the wild apple rhizosphere with geographic and environmental distance was investigated by mantel analysis. The effects of geographic and environmental gradients on microorganisms in the rhizosphere of wild apples were also explored. In this paper, it was hypothesized that geographic and environmental gradients would influence the diversity of microbial communities in the wild apple rhizosphere. To validate this, it was investigated using mantel analysis. In summary, this study offers theoretical guidance for the sustainable management and ecological construction of wild apple forests China.

## Materials and methods

### Site description and sampling

**Fig. 1:**
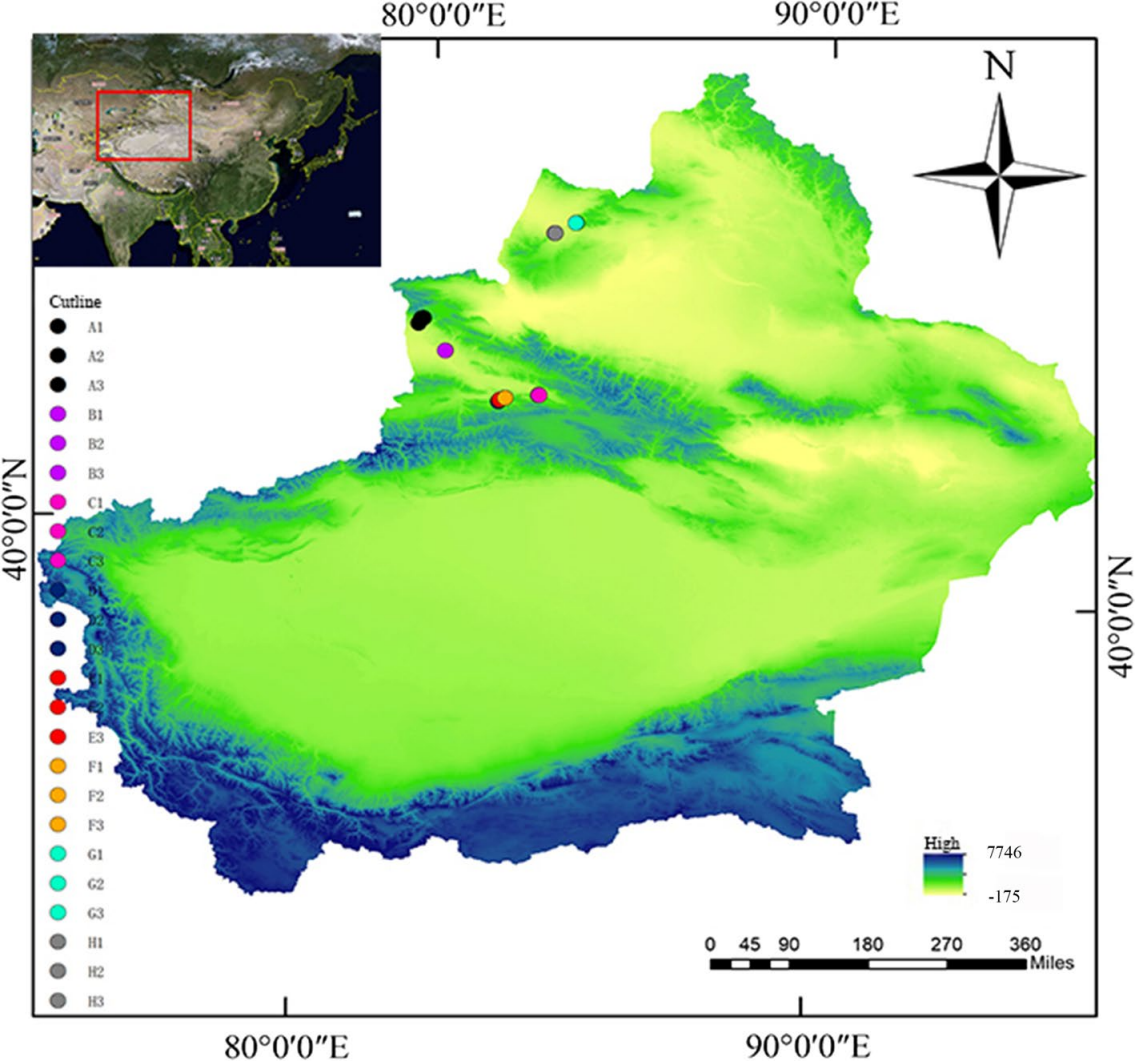
Sampling location and geographical distribution of wild apple rhizosphere soil in Xinjiang. A to H represent Daxigou in Huocheng County, Wild Fruit Forest in Xinyuan County, Resource Nursery, Damorhu Gongliu County, Xiaomoerhu in Gongliu County, Gongliu County Nazi Work Team, Laofengkou Guozigou in Tuoli County, and Yeguolin Scenic Area in Emin County. The numbers represent three replicates of each sample. The digital elevation model was provided by ASTER GDEM – Global Elevation Data

Eight sampling sites (Daxigou in Huocheng County, Wild Fruit Forest in Xinyuan County, Resource Nursery, Damorhu in Gongliu County, Xiaomorhu in Gongliu County, and Gongliu County Nazi Work Team, Laofengkou Guozigou in Tuoli County, and Yeguolin Scenic Area in Emin County) were located in the Yili River Valley and Tacheng region of Xinjiang, China (Fig. 1). The climatic environment of the sample plot was relatively complex. The Ili River Valley has a temperate continental climate, whereas the Tacheng area is in the mid-temperate arid and semiarid regions. Some areas are inhabited by herdsmen year-round, affecting the wild fruit forest due to grazing to varying degrees. Wild apple rhizosphere soil sampling was carried out in May. Within the sampling area, apple trees with a diameter at breast height of 90–100 cm were randomly selected as sample trees. The topsoil was removed with a sterilized spade. The fibrous roots, together with the lateral roots in the 10–20 cm soil layer, were dig out with the soil around them and placed in sterile bags. Samples were collected in three replicates, with multiple points sampled for each replicate. Stones and other debris were picked out and only the 2–3 mm of rhizosphere soil attached to the roots was collected and equal amounts of soil samples were mixed evenly. After collecting the samples, they were divided into two. One was immediately stored in liquid nitrogen for DNA extraction, and the other was used for the determination of the physical and chemical properties of the soil.

### Soil nutrient analysis

Soil moisture content was measured by the drying method. An acidity meter was used to measure soil pH (NYT 1121.2–2006). Soil AP (Available Phosphorus) content was determined by molybdenum-antimony resistance colorimetry (NY/T1121.7–2014). AN (Available Nitrogen) was determined by the diffusion method (DB13/T 843–2007). Soil AK (Available Potassium) was determined by ammonium acetate extraction (NYT 889–2004). The potassium dichromate external heating method (NYT 1121.6–2006) was used to measure soil organic matter (OM). Soil catalase (EA) was measured. Temp (Temperature) and RH(Relative Humidity) were measured with a hygrothermograph (TA621A).

### DNA extraction and high-throughput sequencing

Total genomic DNA of the soil samples was extracted using the CTAB method. DNA concentration was determined by NanoDrop. Purity and completeness were evaluated using 1% agarose gel electrophoresis. DNA was diluted to 1 ng/μl with sterile water. The 16S/18S rDNA genes were amplified using the specific primers with barcodes. The V3 + V4 of 16S and V4 region of 18S were selected amplification and sequencing. The primers used for 16S rDNA gene were 341F (CCTAYGGGRBGCASCAG) and 806R (GGACTCANNGGGTATCTAAT) [14]. The primers for 18S rDNA gene were 528F (GCGGTAATTCCAGCTCCAA) and 706R (AATCCRAGAATTTCACCTCT) [15]. PCR was performed in a 30 μL reaction with 15 μL Master Mix (New England Biolabs), 0.2 μM each of forward and reverse primers and 10 ng of template DNA. The thermocycling conditions were as follows: predegeneration at 98℃ for 1 min, 30 cycles of denaturation at 98 °C for 10 s, annealing at 50 °C for 30 s, and extension at 72℃ for 30 s, followed by a final extension at 72℃ for 5 min. Electrophoretic detection was performed on a 2% agarose gel. Samples with bright main band between 400 and 450 bp were selected for subsequent experiments. PCR products were pooled equally and purified using Kit (Tiangen Biotech). The purified products were used to prepare the library. Sequencing libraries were generated using TIANSeq Fast DNA Library Prep Kit (Tiangen Biotech). Library quality was assessed on a Qubit 2.0 fluorometer (Thermo Scientific) and an Agilent 2100 Bioanalyzer. Finally, the libraries were sequenced on the Illumina platform using a 2 × 250 bp paired-end protocol.

### Amplicon data processing

Bioinformatics analysis of high-throughput sequencing data was performed using QIIME 2, with slight modifications according to the official tutorials. Briefly, the raw sequence data were demultiplexed using the demux plugin. Primers cutting was then performed with the cutadapt plugin [16]. Sequences were quality-filtered, denoised, merged, and chimera-removed using the DADA2 plugin [17]. This was followed by species annotation.

### Statistical analysis

Α-diversity analysis was used to reflect the complexity of the species diversity for the sample by observed-species, Chao1, Shannon, Simpson, ACE, and Good-coverage indices. All indices were calculated by QIIME 2 and displayed with R (version 3.6.2) [18]. β-Diversity analysis was used to assess sample differences in species complexity. β-diversity was calculated for weighted and unweighted Unifrac by QIIME 2. Principal component analysis (PCoA) was performed prior to cluster analysis. The dimension of the original variables was decreased using the “statpackage” and “ggbiplot” packages. The three-dimensional PCoA results were displayed using QIIME 2, while the two-dimensional results were displayed using the “ade” and “ggplot2” packages in R, Metastats and STAMP were utilized to confirm differences in the abundance of individual taxonomic or functional annotations between the groups. Linear discriminate analysis (LDA) effect size (LEfSe) was used the potentially enriched microbial lineages with significant difference (*P* < 0.05) between different groups at various taxonomic levels with LDA threshold set ≥ 4. Analysis of variance (ANOVA) was performed the differences between the two groups of microbial communities based on the Bray–Curtis dissimilarity distance matrix [18, 19]. In addition, the correlations between un Weighted or Weighted UniFrac distance matrices and the spatial distance matrices were measured by using partial Mantel test in R.

## Results

### Diversity of 16S/18S rDNA genes in different regions

**Fig. 2:**
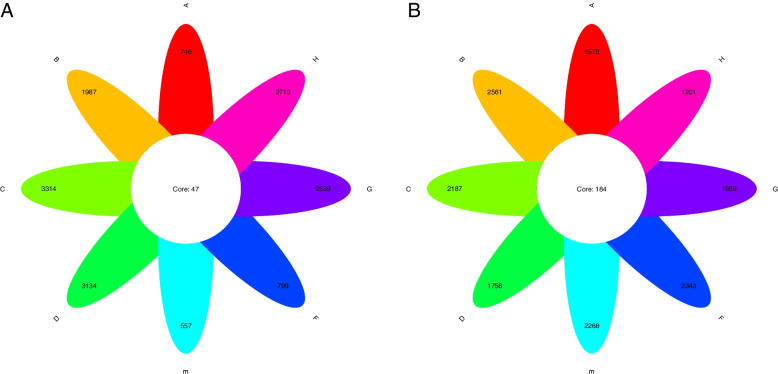
Petal plot based on OTUs: (**a**) bacterial (**b**) eukaryotic. Different colored petals represent the number of OTUs of samples from different regions, and the number of cores represents the number of OTUs common to all samples. A to H represent Daxigou in Huocheng County, Wild Fruit Forest in Xinyuan County, Resource Nursery, Damorhu in Gongliu County, Xiaomorhu in Gongliu County, Gongliu County Nazi Work Team, Laofengkou Guozigou in Tuoli County, and Yeguolin Scenic Area in Emin County

**Fig. 3:**
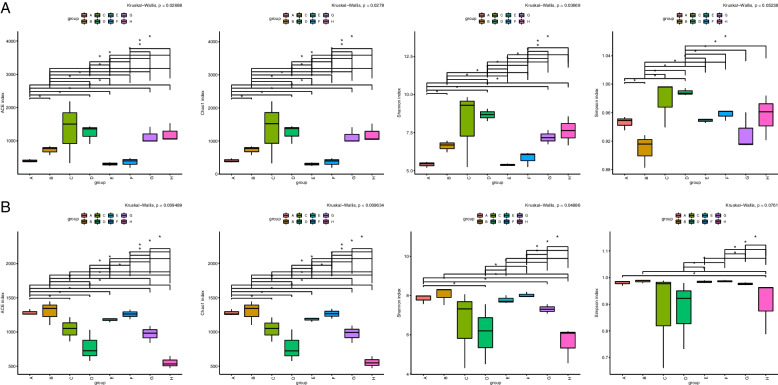
Cluster analysis results in sequence number and diversity/richness index of 97%. (**a**) bacterial (**b**) eukaryotic. Different colors represent regions A to H

16S/18S rDNA genes sequencing was used to study the population characteristics of rhizosphere bacteria and eukaryotes in wild apples from different regions of Xinjiang. After filtering, denoising, and removing chimeras, the average number of bacterial sequences per sample was 54,992, with an effective rate of 79.26%. The average number of eukaryotic sequences was 108,548, with an effective rate of 84.79%. The bacterial sequences were clustered into 15,789 operational taxonomic units (OTUs), and eukaryotic sequences were clustered into 16,853 OTUs. The coverage of all samples was > 0.99, and with the rarefaction and Shannon curves converged. This indicates that sampling was reasonable, and that the sequencing was sufficient to characterize the diversity of the study sites. A plot shows (Fig. 2) that there were 47 core OTUs of bacteria and 184 core OTUs of eukaryotes, respectively, in wild apples rhizosphere soil samples from eight regions in Xinjiang. The number of bacterial OTUs in regions C, D, G, and H was greater than that of eukaryotic OTUs, whereas the number of bacteria in regions A, B, E, and F was lesser than that of eukaryotic OTUs. The results showed that the number of OTUs in each soil sample varied across the eight regions. The bacterial Chao 1, ACE, Shannon and Simpson indices in regions C, D, G, and H were higher than the eukaryotic biodiversity indices, whereas the bacterial diversity indices in regions A, B, E, and F were lower than the eukaryotic biodiversity indices (Fig. 3). The α-diversity analysis showed that species diversity in different regions was quite different. The bacterial species diversity in regions C, D, G, and H was higher than in other regions whereas regions A, B, E, and F had higher species diversity than in other regions.

**Fig. 4:**
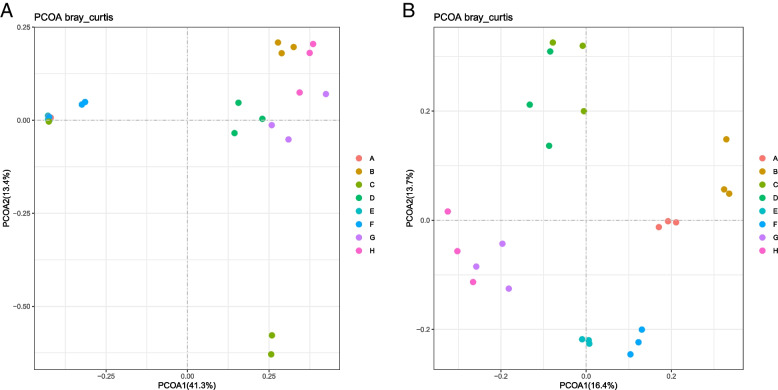
OTU PCoA based on Bray–Curtis distance method: (**a**) bacterial (**b**) eukaryotic. Different colors represent samples from different regions, A to H represent different regions

PCoA was used to rank at OTU level to reveal similarities or differences in community structure between different regional groups. The first and second axes of the bacterial community structure contributed 41.3% and 13.4% of the explanation, respectively, and 16.4% and 13.7% for eukaryotes, respectively. (Fig. 4a,b). In general, the majority of samples from each group clustered together, indicating significant differences in the community composition of bacterial and eukaryotic species.

### Rhizosphere bacteria species composition in different regions

**Fig. 5:**
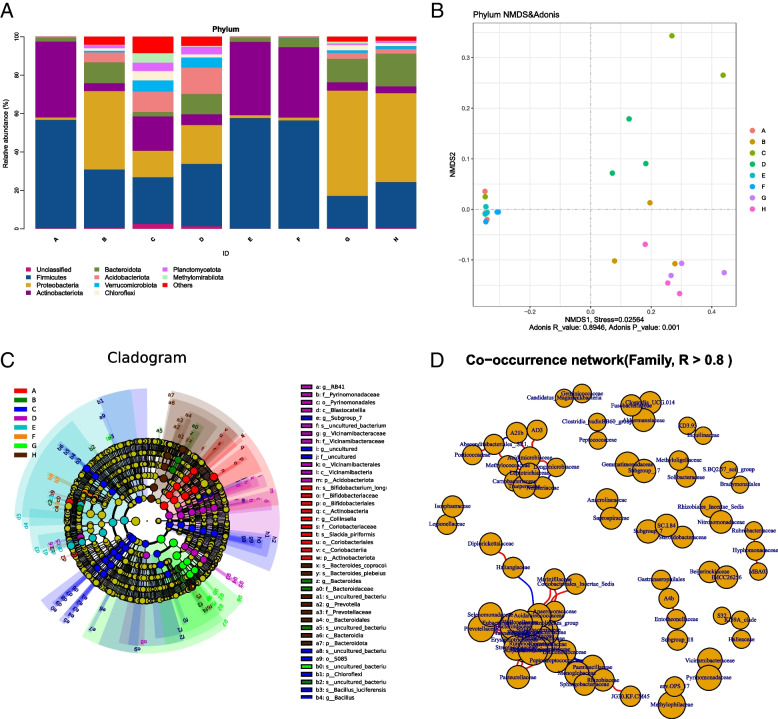
Composition of 16S rDNA species communities. **a** Relative abundance of bacterial communities at the phylum level. **b** Nonmetric multidimensional scale (NMDS) ordering of ASV-level data. **c** Clade map of LEfSe analysis of major bacterial differential microbiota in eight regions. Each small circle on a different classification level represents a classification at that level, and the diameter of the small circle is proportional to the relative abundance. The coloring principle is to uniformly color the species with no significant difference as yellow and the other species with differences according to the species. The most abundant groupings are colored. **d** Bacterial association network analysis diagram. The nodes represent individual family, and the size of the nodes represents the average relative abundance in the sample. The connections between the nodes indicate a correlation between the two family, the red line indicates a positive correlation, and the blue line indicates a negative correlation. The thickness of the line is proportional to the correlation between family, and the thicker the line, the stronger is the correlation. At the same time, the more connections through a node, the more closely related the family are to other members of the flora

Compared to the Silva138 database, the proportion of phylum, class, order, family, genus, and species in bacteria was 97.78%, 97.24%, 95.8%, 94.24%, 84.63%, and 32.39% respectively. The 16S rRNA gene sequences were divided into phyla, and the nine most abundant phyla were Firmicutes, Proteobacteria, Actinobacteriota, Bacteroidota, Acidobacteriota, Verrucomicrobiota, Chloroflexi, Planctomycetota, and Methylomirabilota, unclassified (Fig. 5a). Firmicutes were the most dominant bacterial phylum in regions of A (56.47%), C (24.46%), D (32.45%), E (57.58%), and F (56.43%), and Proteobacteria were the most dominant bacterial phylum in regions B (40.82%), G (54.70%), and H (46.23%). The community composition in regions A, E, and F was less; in particular, the relative abundance of Acidobacteriota, Verrucomicrobiota, Planctomycetota, Chloroflexi, and Methylomirabilot was very low. The relationships between the sequential datasets were visualized by non-metric multidimensional scale (NMDS), and the samples in each region were basically clustered together, but the distance between groups was large, with a *P* ≤ 0.001 (Fig. 5b), indicating differences in the composition of bacterial communities in different regions.

Linear discriminant analysis effect size (LEfSe) analysis was used to identify microorganisms explicitly enriched in bacteria from the phyla-to-species level in different regions. The results showed that there were 41 phylum-to-species differences in the eight regions. In the bacterial species community, the various species in region A were Bifidobacteriaceae, Actinobacteria, and Coriobacteriales. The different communities in region B were Bacteroidaceae and uncultured_bacteria. Chloroflexi, *Bacillus*_*luciferensis*, and uncultured bacteria were the different species in region C. The main communities in region D included Vicinamibacteraceae, Blastocatellia, and Pyrinomonadaceae. There were no apparent species differences in regions E and F, but uncultured_bacteria were different in region G. *Prevotella* and Bacteroidia were different in region H (Fig. 5c). Figure 5d shows that the rhizosphere bacterial family of wild apples co-occurred. The results showed that the rhizosphere bacterial communities of wild apples in different regions cooperated, and a few competed.

### Rhizosphere eukaryotic species composition in different regions

**Fig. 6:**
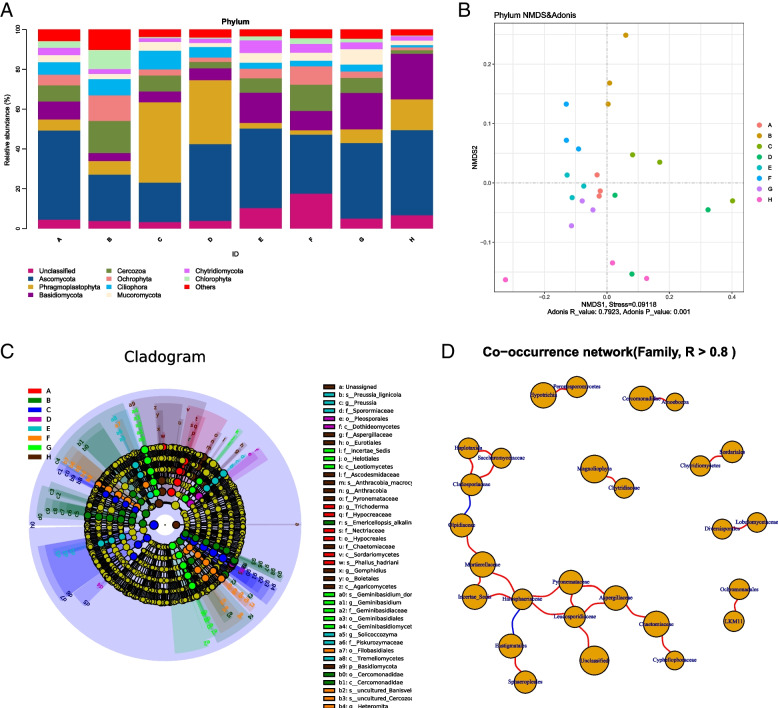
Composition of 18S rDNA species communities. **a** Relative abundance of eukaryotic communities at the phylum level. **b** Nonmetric multidimensional scale (NMDS) ordering of ASV-level data. **c** Clade map of LEfSe analysis of the significant differential microbiota of eukaryotes in the eight regions. Each small circle on a different classification level represents a classification at that level, and the diameter of the small circle is proportional to the relative abundance. The coloring principle is to uniformly color the species with no significant difference as yellow, and the other species with differences according to the species. The most abundant groupings are colored. **d** Diagram of eukaryotic association network analysis. The nodes represent individual species, and the size of the nodes represents the average relative abundance in the sample. The connections between the nodes indicate a correlation between the two species, the red line indicates a positive correlation, and the blue line indicates a negative correlation. The thickness of the line is proportional to the correlation between species, and the thicker the line, the stronger is the correlation. At the same time, the more connections through a node, the more closely related the species are to other members of the flora

The proportion of the eukaryotic phylum level was 83.17%, that of the class level was 75.23%, that of the order-level was 69.39%, that of the family level was 61.79%, that of the genus level was 53.09%, and that of the species-level was 30.05%. The 18S rRNA gene sequences were divided into phylum levels, among which the nine most abundant phyla were Ascomycota, Phragmoplastophyta, Basidiomycota, Cercozoa, Ochrophyta, Ciliophora, Mucoromycota, Chytridiomycota, and Chlorophyta, unclassified (Fig. 6a). Eukaryotes were dominated by Ascomycota, Phragmoplastophyta, and Basidiomycota. Ascomycota was the most dominant phylum in regions A (44.71%), B (23.23%), D (38.59%), E (39.98%), F (29.58%), G (37.94%) and H (42.70%), and Phragmoplastophyta was the most dominant phylum in region C (40.42%) regions. NMDS analysis showed that the samples in region C were far apart, and the samples in each area were clustered together, with a *P* ≤ 0.001 (Fig. 6b), indicating differences in the eukaryotic community composition in different regions.

LEfSe analysis showed significant differences in the leading 41 phyla-to-species in the eight different regions. In the eukaryotic community, the main differential species in region A were *Trichoderma*, Nectriaceae, and *Phallus_hadriani*. The differential species in region B was Cercomonadidae, and there was no apparent difference in fungal species. In region C, there was also no significant difference in species. Pleosporales and Dothideomycetes were the differential species in region D. *Solicoccozyma* and Piskurozymaceae were the differential species in region E. The differential species in region F included Filobasidiales. Helotiales, Incertaesedis, Leotiomycetes, and *Geminibasidium* were the differential species in region G (Fig. 6c). Figure 6d shows that the rhizosphere eukaryotic community of wild apples co-occured, and the network structure was different from that of bacterial. The results showed that the rhizosphere eukaryotic communities of wild apples in different regions cooperated with each other, and a few competed.

### Relationship between β-diversity and environmental factors

**Fig. 7:**
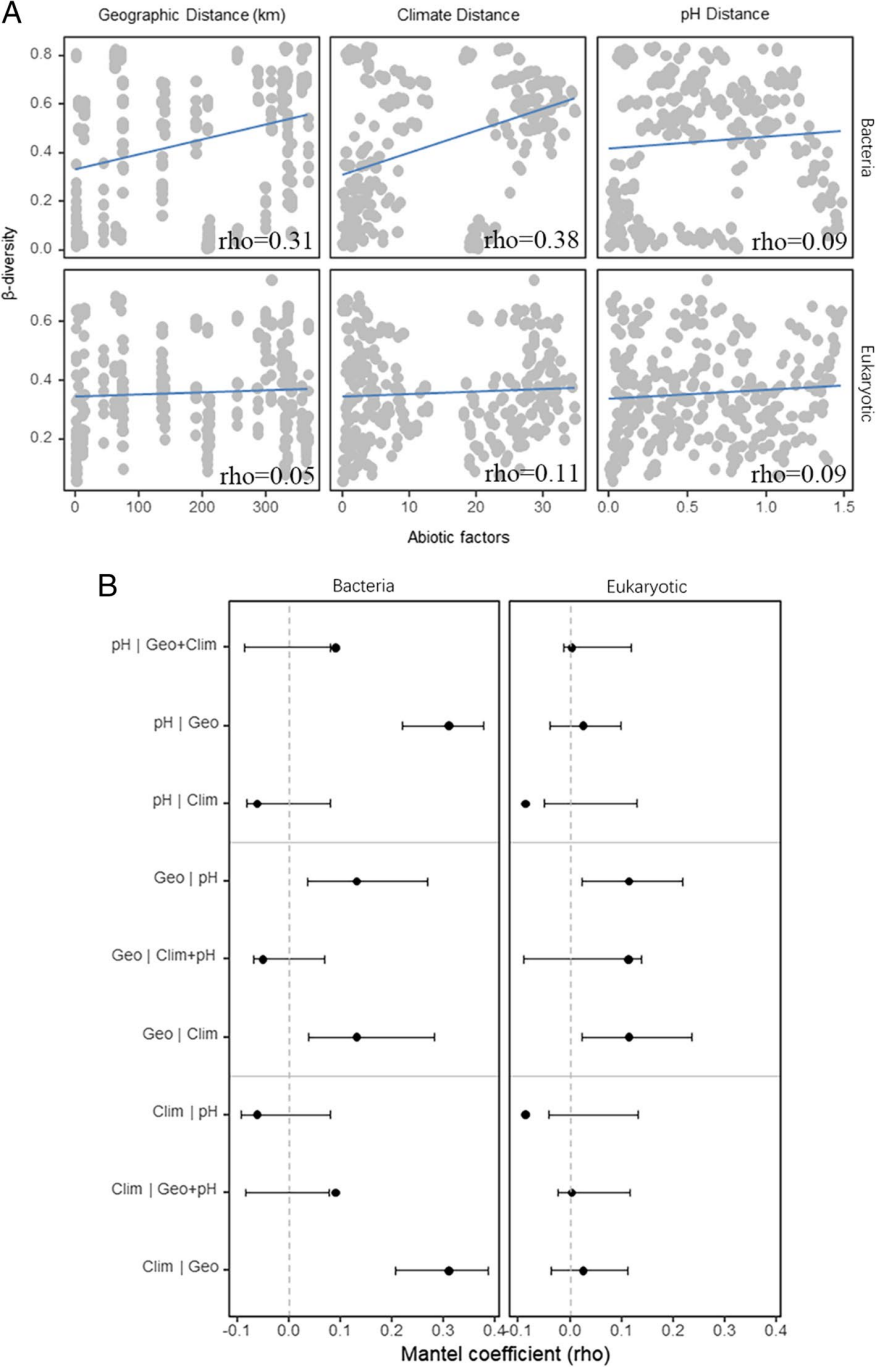
Relationships between environmental factors and bacteria and eukaryotic β-diversity. **a** Bivariate associations between β-diversity and environmental factors (geographic, climatic, and soil pH distances). Lines represent the trends of the bivariate associations. Spearman correlation coefficients (rho) and the lower and upper 95% confidence intervals of simple Mantel tests are shown. **b** Partial Mantel tests for the bivariate associations between environmental factors and β-diversity. Symbol | represents partial Mantel tests. Variables in the left of | represent the independent variables, and in the right of | represent the controlling independent variables. Points represent the Spearman correlation coefficients (rho) and error bars 95% confidence intervals. Geo—geographic distance; Clim—climatic distance; pH—soil pH distance. Geographic distance has a unit of km, and the other distance metrics are unitless

The impact of geographic distance, climatic distance and soil pH on the composition of rhizosphere bacterial and eukaryotic communities in wild apples was examined by mantel analysis. It was found that the correlation between climatic distance and β-diversity was greater than that between geographic distance and soil pH (Fig. 7a). Geographic and climatic distances correlate were more strongly correlated with bacterial β-diversity than with eukaryotic β-diversity. Geographic distance remained positively correlated with eukaryotic β-diversity even after controlling for climatic distance and/or soil pH (Fig. 7b). The results indicated that geographic and climatic differences were important predictors for microbial β-diversity. Soil pH and climatic distance were positively with bacterial β-diversity and insignificantly with eukaryotic β-diversity when geographic distance was controlled (Fig. 7b). Climate distance was negatively with microbial β-diversity when the soil pH was controlled. The converse was also true. In summary, there was a correlation between geographical distance, climatic distance and soil pH with microbial β-diversity.

## Discussion

### Rhizosphere microbial diversity in different regions

The microbial balance in the rhizosphere of wild apple forests in Xinjiang was one of the main driving forces shaping ecosystem function. It contributed to the “soil-microbe-plant” nutrient cycle, primary production and waste decomposition [20]. Therefore, it was crucial to understand the structure, diversity and functional role of microbial communities in terms of community composition, functional groups and ecosystems when human activities, such as climate change, pest and disease outbreaks and grazing, affected the balance of microorganisms in the rhizosphere [20]. This study explored the biodiversity, community structure and functional prediction of wild apples rhizosphere bacteria and eukaryotes in Xinjiang. The results showed that there were more bacterial OTUs than eukaryotic OTUs in zones C, D, G, and H, and the number of bacterial OTUs in the other zones decreased to varying degrees. While the number of eukaryotic OTUs in regions D, G, and H was lesser than that in other regions (Fig. 2). Although the species diversity of different regions was quite different, overall, in the α-diversity study, the bacterial species diversity in regions C, D, G, and H was higher than that of eukaryotic species, and the evenness of species distribution was also greater than that of eukaryotic species. Regions A, B, E, and F showed the opposite results. Blaire Steven et al. (2021) suggested that the fir tree rhizosphere microbial 16S rRNA gene dataset was more diverse than the 18S rRNA gene dataset, and the bacterial community was more varied than the eukaryotes community [7]. The diversity study results were consistent with the rhizosphere microbial diversity conclusions in regions C, D, G, and H. However, they were in contrast to the diversity study results in regions A, B, E, and F. According to Yurong Yang et al. (2021), regarding the microbial diversity and communities in the rhizosphere and inner compartments of grassland dominant perennials, grazing decreased interactions among bacterial genera, but there was no difference in interactions among fungal genera [21]. Their results were similar to those of regions A, B, E, and F. Therefore, the differences in microbial diversity in the rhizosphere of wild apples in different regions of Xinjiang might be due to the decrease in the number of microorganisms in regions A, B, E, and F, owing to different climates, grazing, and loss of soil and water nutrients, resulting in greater regional eukaryotes than bacteria.

In this study, the dominant bacteria taxa in wild apples rhizosphere soil included Firmicutes, Proteobacteria, Actinobacteriota, Bacteroidota, Acidobacteriota, Verrucomicrobiota, Chloroflexi, Planctomycetota and Methylomirabilota (Fig. 5a). The bacterial community composition in regions A, E, and F was less, and the relative abundance of Acidobacteriota, Verrucomicrobiota, Chloroflexi, Planctomycetota, and Methylomirabilota was low. The bacterial community composition of region B was similar to that of regions C, D, G, and H, but its OTU was lower. The bacterial community composition differed significantly in the eight regions. Firmicutes are primarily endospore-forming bacteria, and one of the most abundant and ubiquitous bacterial groups in the environment, surviving grazing, fertilization [22], and harsh [23] environments [24]. Alexandre suggested that the conversion of forest to pasture increased the abundance of microbial taxa associated with nitrogen fixation, including Bacteroidetes and Firmicutes [25]. The relative abundance of Firmicutes in the rhizosphere soil of regions A and C to F regions was relatively high, indicating that the number of *Firmicutes* in regions A and C to F may have increased due to grazing and adverse climatic conditions. Proteobacteria are one of the rhizosphere trophic bacteria, that promote nitrogen cycling [26, 27]. The relative abundance of Proteobacteria in regions B, G, and H was high, but the relative abundance in other regions was low. It was speculated that the number of Proteobacteria was high in these three regions due to the lack of grazing or abandonment of grazing, consistent with the results obtained by Alexandre Pedrinho [25]. Verrucomicrobia and Acidobacteriota were thought to survive in nutrient-limited soil environments [28]. The relative abundance of the two bacteria in regions D, G, and H was higher, but some soil nutrients in these regions were higher than those in other regions (Table 1). Therefore, the local climatic environment and other nutrients in the soil should be considered comprehensively.

**Table 1:** Soil nutrients of wild apples in Xinjiang in different regions

Sample group	pH	OM(g/kg)	AN (mg/kg)	AP (mg/kg)	AK (mg/kg)	Moisture (%)	EA(IU)	RH (%)	Temp (℃)
A1	7.21 ± 0.01ij	55.67 ± 0.12e	418.02 ± 0.91a	7.52 ± 0.29 mn	314.61 ± 0.26j	26.05 ± 0.20f	8.84 ± 0.01c	60.45 ± 1.94a	19.79 ± 1.76b
A2	7.18 ± 0.01j	55.47 ± 0.28e	383.72 ± 2.03b	12.16 ± 0.25 l	318.96 ± 1.60j	26.04 ± 0.16f	8.78 ± 0.01d	60.45 ± 1.94a	19.79 ± 1.76b
A3	7.22 ± 0.04ij	55.63 ± 0.13e	424.20 ± 2.1a	8.15 ± 0.34 m	316.91 ± 3.38j	25.28 ± 0.04f	8.77 ± 0.01d	60.45 ± 1.94a	19.79 ± 1.76b
B1	8.27 ± 0.05a	10.27 ± 0.03 l	81.32 ± 3.29o	25.15 ± 0.35e	161.21 ± 0.68 k	13.75 ± 0.23 m	7.46 ± 0.02 k	57.31 ± 0.74ab	21.70 ± 0.59ab
B2	8.25 ± 0.04a	10.40 ± 0.10 l	124.37 ± 0.91 m	28.04 ± 0.78d	161.47 ± 0.51 k	14.92 ± 0.26 l	7.23 ± 0.01 m	57.31 ± 0.74ab	21.70 ± 0.59ab
B3	8.24 ± 0.03a	10.27 ± 0.33 l	97.42 ± 0.91n	32.50 ± 0.40c	160.44 ± 0.92 k	13.34 ± 0.22 m	7.34 ± 0.01 l	57.31 ± 0.74ab	21.70 ± 0.59ab
C1	7.31 ± 0.03ghi	44.23 ± 0.15f	346.15 ± 3.16d	22.89 ± 0.30f	604.03 ± 0.92f	44.55 ± 0.10de	8.30 ± 0.01i	60.81 ± 2.58a	19.72 ± 1.17b
C2	7.27 ± 0.03hij	43.87 ± 0.15f	377.65 ± 6.89b	17.92 ± 0.44ij	623.71 ± 3.26e	44.18 ± 0.17e	8.32 ± 0.01i	60.81 ± 2.58a	19.72 ± 1.17b
C3	7.42 ± 0.02 g	44.23 ± 0.23f	363.18 ± 4.04c	18.37 ± 0.45i	620.39 ± 0.77e	45.59 ± 0.18bc	8.41 ± 0.01 h	60.81 ± 2.58a	19.72 ± 1.17b
D1	8.12 ± 0.04bc	38.27 ± 0.12gh	137.20 ± 1.21 l	16.34 ± 0.54jk	438.10 ± 0.68 h	17.93 ± 0.20i	8.21 ± 0.01j	51.81 ± 0.99b	21.93 ± 1.41ab
D2	8.20 ± 0.03ab	38.53 ± 0.23 g	126.35 ± 1.95 m	22.95 ± 0.39f	438.86 ± 0.51 h	17.02 ± 0.72j	8.18 ± 0.01j	51.81 ± 0.99b	21.93 ± 1.41ab
D3	8.09 ± 0.03 cd	38.10 ± 0.06 h	149.57 ± 2.76 k	15.60 ± 0.59 k	439.38 ± 1.33 h	19.15 ± 0.22 h	8.20 ± 0.02j	51.81 ± 0.99b	21.93 ± 1.41ab
E1	8.00 ± 0.02de	35.13 ± 0.03 k	258.65 ± 0.93f	20.75 ± 0.35gh	342.22 ± 0.26i	45.04 ± 0.31 cd	8.67 ± 0.02f	53.49 ± 1.35b	23.08 ± 1.50ab
E2	8.00 ± 0.07de	34.83 ± 0.12 k	218.98 ± 2.83gh	20.41 ± 0.61 h	346.06 ± 0.51i	46.34 ± 0.48b	8.71 ± 0.01e	53.49 ± 1.35b	23.08 ± 1.50ab
E3	7.91 ± 0.04e	35.23 ± 0.19 k	251.30 ± 2.38f	22.33 ± 0.65 fg	342.73 ± 1.17i	45.13 ± 0.10 cd	8.70 ± 0.02ef	53.49 ± 1.35b	23.08 ± 1.50ab
F1	7.41 ± 0.08 g	77.73 ± 0.12a	224.93 ± 3.54 g	6.28 ± 0.72no	1811.53 ± 5.11a	45.61 ± 0.34bc	9.14 ± 0.00ab	52.60 ± 1.18b	23.83 ± 0.67ab
F2	7.37 ± 0.03gh	77.30 ± 0.10b	255.85 ± 2.33f	8.71 ± 0.43 m	1780.85 ± 9.22b	47.99 ± 0.10a	9.17 ± 0.01a	52.60 ± 1.18b	23.83 ± 0.67ab
F3	7.67 ± 0.03f	77.80 ± 0.17a	300.65 ± 3.51e	5.60 ± 0.49o	1785.96 ± 11.72b	48.09 ± 0.09a	9.12 ± 0.01b	52.60 ± 1.18b	23.83 ± 0.67ab
G1	6.93 ± 0.03 k	36.73 ± 0.12i	156.22 ± 2.16 k	40.35 ± 0.89a	456.76 ± 0.26 g	21.67 ± 0.44 g	6.82 ± 0.01o	28.63 ± 2.88c	25.45 ± 2.20a
G2	6.89 ± 0.03kl	36.20 ± 0.17j	197.63 ± 1.72i	35.61 ± 0.99b	450.37 ± 1.11 g	21.96 ± 0.11 g	7.25 ± 0.02 m	28.63 ± 2.88c	25.45 ± 2.20a
G3	6.88 ± 0.02kl	36.97 ± 0.12i	181.77 ± 4.47j	29.17 ± 0.64d	449.60 ± 051 g	19.79 ± 0.17 h	7.05 ± 0.01n	28.63 ± 2.88c	25.45 ± 2.20a
H1	6.84 ± 0.03kl	56.53 ± 0.03d	215.60 ± 2.05	18.60 ± 1.24i	648.26 ± 0.26d	16.01 ± 0.15 k	8.47 ± 0.01 g	25.98 ± 2.57c	25.39 ± 2.40a
H2	6.81 ± 0.0.1 l	56.23 ± 0.15d	224.70 ± 1.93 g	30.97 ± 0.73c	673.06 ± 0.92c	17.45 ± 0.36ij	8.73 ± 0.01e	25.98 ± 2.57c	25.39 ± 2.40a
H3	6.79 ± 0.02 l	57.03 ± 0.09c	256.32 ± 2.71f	21.93 ± 0.74fgh	664.87 ± 1.33c	17.02 ± 0.32j	8.70 ± 0.01ef	25.98 ± 2.57c	25.39 ± 2.40a

Different letters indicate significant differences (*P* < 0.05) of soils based on a one-way ANOVA followed by an LSD test

The eukaryotic community composition included Ascomycota, Phragmoplastophyta, Basidiomycota, Cercozoa, Ochrophyta, Ciliophora, Mucoromycota, Chytridiomycota, and Chlorophyta (Fig. 6a). The eukaryotic community composition was more uniform and less variable than the bacterial community composition. Ascomycota and Basidiomycota are essential decomposers in the rhizosphere; they promote nutrient absorption, have a high tolerance to environmental stress [29, 30], and were distributed in the eight regions of our study. Cercozoa is a bacterial predator [31] with a higher relative abundance in regions B and F. Blaire Steven et al. also found Cercozoa in the fir tree rhizosphere and was sensitive to changes in soil pH [7]. Chytridiomycota was associated with cellulose degradation [32], was distributed in all the eight regions of our study, and was more abundant in region E. Overall, the changes in bacterial community composition in different regions were more significant than those in eukaryotic community composition. In addition, NMDS analysis, LEfSe analysis, and network analysis indicated changes in the rhizosphere microbial community structure in different regions. LEfSe analysis showed significant differences among the rhizospheres in different regions. These differences were probably caused by regional climate change, human activities, and various environmental factors on microorganisms [33], indicating that the rhizosphere soil environment of 10 to 20 cm was affected by regional ecological changes. The stability of the rhizosphere ecological environment can also be analyzed through the symbiotic network. The number of bacterial community connections was higher than that of eukaryotic community connections, and the microbial communities cooperated with each other, indicating that the rhizosphere environment composed of the overall microbial community was relatively stable.

### Factors affecting β-diversity in wild apples of different regions

Many studies demonstrated that environmental factors had important impacts on the abundance, structure and function of microbial communities [34]. In this study, correlations between differences in geographic distance, climatic distance and soil pH with bacterial as well as eukaryotic community composition were tested by mantel analysis. Meanwhile, the effects of geographic and environmental gradients on wild apple rhizosphere microbes were explored. A study by Shi et al. on *Fritillaria thunbergii* showed that soil pH had an effect on bacterial species composition [35]. This was consistent with the results of the present work, that is, soil pH was positively with bacterial β-diversity, controlling for geographic distance. However, in this study, there was no significant correlation between soil pH and eukaryotic β-diversity. In contrast, a study by Ren et al. [36] found that the abundance and community structure of eukaryotic OTUs in lake sediments correlated with soil pH. Differences in microbial communities were due to a combination of plant and ecological influences and it was difficult to identify a single factor that had an impact. Therefore, it was normal for the results of individual studies to differ. In addition, Jing et al. [37] and Na et al. [38] confirmed that geographic and climatic distance were significantly associated with microbial community. This was consistent with the findings of this study. That is, rhizosphere microbial β-diversity was correlated with geographic and climatic distance. Among these, bacterial β-diversity showed a greater correlation with geographic and climatic distance than eukaryotes. It will help to maintain the stability of the wild apple rhizosphere and conserve its germplasm resources by understanding the interactions between microbial communities and geographic and climatic distances.

In conclusion, this study revealed the structure and diversity rhizosphere microbial communities from eight sampling regions where wild apples were mainly distributed, and their relationship with geographic and environmental gradients. The results demonstrated that the rhizosphere microbial community were affected to certain extent by geographic distance, climate change and grazing. These factors reduced the stability of the rhizosphere. For future research, the effect of grazing on rhizosphere microorganisms at different depths of as well as the interaction mechanism between wild apples and rhizosphere microorganisms can be further explored. It is a way of conserving wild apple germplasm resources.

## Conclusion

In summary, the stability of the rhizosphere environment wild apples in Xinjiang was closely related to geographic distance, climate change, and human activities. The bacterial OTUs numbers, Shannon values, and community composition were significantly lower in regions A, E, and F. The abundance of Acidobacteriota, Verrucomicrobiota, Planctomycetota, Chloroflexi, and Methylomirabilota was also low in these regions. The community composition of region B was similar to that of regions C, D, G, and H, but the number of OTUs and Shannon values were lower than in these four regions with higher values than in regions A, E, and F. The results showed that the abundance and diversity of wild apples rhizosphere bacteria in regions A, B, E, and F were relatively low and were greatly affected by the external environment. However, there were differences in the relative abundance of eukaryotic communities, and they were less than those of bacterial communities. In addition, mantel analysis showed that geographic and climatic distance were key factors influencing the composition and diversity of wild apple rhizosphere microbial communities. While these two factors correlated more with bacterial diversity than that of eukaryotes. Furthermore, this study confirmed the relevance of the structure and diversity of wild apple rhizosphere microbial community to geographic and environmental gradients. The results of this study provide a theoretical basis for subsequent research on microbial function mining and biofertilizer. Further, it can serve as a theoretical guide for regulating the stable balance of wild apple rhizosphere microbial communities and the sustainable management of wild apple forests.

## Data Availability

Data of this project have been deposited in the Global Biodiversity Information Facility (GBIF). Repository name: Malus sieversii (Ldb.) Roem. Rhizosphere Microorganism Database. Data identification number: 10.15468/dd.f9zy25 (https://www.gbif.org/derivedDataset/10.15468/dd.f9zy25).
